# “Unspecified Dissociative Disorder: A Case Report”

**DOI:** 10.1192/j.eurpsy.2025.1187

**Published:** 2025-08-26

**Authors:** F. J. Gonzalez Zapatero, L. Del Canto Martinez, M. B. Arribas Simon, P. Martinez Gimeno, B. Rodriguez Rodriguez, M. A. Andreo Vidal, M. Rios Vaquero, A. Monllor Lazarraga, M. P. Pando Fernandez, M. Calvo Valcarcel, N. Navarro Barriga, M. Fernandez Lozano, M. J. Mateos Sexmero, L. Rojas Vazquez

**Affiliations:** 1 Hospital Clinico Universitario de Valladolid, Valladolid, Spain

## Abstract

**Introduction:**

Psychosis is a complex mental disorder characterized by a profound impairment in reasoning, emotional regulation, memory, and daily functioning. It is often accompanied by symptoms such as delusions and hallucinations, which can significantly disrupt an individual’s ability to engage in everyday activities. This case study focuses on a 37-year-old Senegalese woman who presented with disorganized behavior and incoherent speech, illustrating the challenges in diagnosing and treating unspecified psychosis.

**Objectives:**

The primary aim of this study is to analyze the clinical presentation, differential diagnosis, and treatment outcomes of a patient diagnosed with unspecified psychosis. Additionally, it seeks to highlight the importance of cultural considerations and language barriers in psychiatric assessment.

**Methods:**

The patient arrived at the emergency department in a distressed state, accompanied by a friend due to her inability to communicate in Spanish. Clinical assessment revealed disorganized speech, severe anxiety, and potential auditory hallucinations. The clinical interview indicated that the patient had exhibited increasingly erratic behavior over several weeks, including withdrawing from work and engaging in bizarre actions such as throwing objects and speaking to herself. Comprehensive laboratory tests, including routine blood work and urine analysis, were conducted to exclude other psychiatric and medical conditions.

**Results:**

The laboratory results were largely normal, with the exception of a positive serology for hepatitis B. A thorough differential diagnosis ruled out other psychiatric disorders, including schizophrenia, substance-induced psychosis, and major depressive episodes. After establishing the diagnosis of unspecified psychosis, the treatment regimen was initiated with Amisulpride (100 mg) and Lorazepam (1 mg). Following treatment, the patient demonstrated a significant reduction in delusional thoughts and an improvement in her overall psychiatric condition.

**Conclusions:**

The findings support the effectiveness of atypical antipsychotic medication in treating unspecified psychosis, particularly in alleviating delusional ideas and associated psychiatric symptoms. Furthermore, this case underscores the critical role of family support and the necessity for ongoing monitoring and follow-up in the management of psychotic disorders. It emphasizes the need for culturally sensitive approaches to psychiatric care, especially in cases involving language barriers.

This case illustrates the complexities involved in diagnosing and treating unspecified psychosis, particularly in patients from diverse cultural backgrounds. Early intervention and a combination of pharmacological and psychosocial support are essential for improving patient outcomes. Continued research and awareness are necessary to enhance the understanding and management of psychotic disorders in various populations.

**Disclosure of Interest:**

None Declared

